# Hypothalamic AMPK as a Regulator of Energy Homeostasis

**DOI:** 10.1155/2016/2754078

**Published:** 2016-07-28

**Authors:** My Khanh Q. Huynh, Ann W. Kinyua, Dong Joo Yang, Ki Woo Kim

**Affiliations:** Departments of Pharmacology and Global Medical Science, Wonju College of Medicine, Yonsei University, Wonju 26426, Republic of Korea

## Abstract

Activated in energy depletion conditions, AMP-activated protein kinase (AMPK) acts as a cellular energy sensor and regulator in both central nervous system and peripheral organs. Hypothalamic AMPK restores energy balance by promoting feeding behavior to increase energy intake, increasing glucose production, and reducing thermogenesis to decrease energy output. Besides energy state, many hormones have been shown to act in concert with AMPK to mediate their anorexigenic and orexigenic central effects as well as thermogenic influences. Here we explore the factors that affect hypothalamic AMPK activity and give the underlying mechanisms for the role of central AMPK in energy homeostasis together with the physiological effects of hypothalamic AMPK on energy balance restoration.

## 1. Introduction

The central nervous system (CNS) plays an important role in energy balance maintenance by regulating energy intake, energy expenditure, and energy storage [1]. The energy obtained from food intake is used for regular physiological activities in the whole body including heat production through metabolism and the excess energy is stored in form of fat, glycogen, and protein [2]. In order to maintain a functional energy homeostasis, it is important to have a balance between energy intake and energy expenditure. Excessive food intake resulting from dysregulated appetite and impaired energy expenditure is among the factors disrupting energy balance [3]. The arcuate nucleus (ARC) of the hypothalamus is composed of neuronal populations responding to orexigenic and anorexigenic neuropeptides and acts as the primary appetite control center [4–6]. In the ARC, activation of the orexigenic neuropeptides, neuropeptide Y (NPY), and agouti-related protein-expressing neurons (AgRP) results in increased food intake while activation of the anorexigenic neuropeptide proopiomelanocortin (POMC) in satiety conditions suppresses food intake [7]. The energy consumed from food is broken down in metabolic processes to make carbon dioxide, water, and heat. Under resting condition, the body releases energy in form of heat and this output of energy is referred to as thermogenesis [8]. When the body is exposed to cold temperature, the sympathetic nervous system is activated via the *β*-adrenergic receptor resulting in increased thermogenesis [9].

An imbalance in energy consumption and energy expenditure stimulates the CNS and the peripheral metabolic system to initiate metabolic processes in order to restore the energy balance. In case of increased energy intake, the brain suppresses feeding behavior or stimulates storage of the excess energy in other tissues such as glycogen in liver or triglycerides in adipose tissue. On the other hand, increased energy expenditure in comparison to energy intake stimulates appetite and inhibits energy expenditure through various metabolic pathways including fatty acid metabolism and activation of the nutrient sensor AMP-activated protein kinase (AMPK) [10]. AMPK is activated by high ratio of ADP : ATP or AMP : ATP and is regarded as a key player in the peripheral and central energy regulation [11]. Over the past few years many studies have focused on understanding the underlying mechanisms involved in AMPK mediated energy homeostasis regulation.

AMPK, which is a serine threonine kinase comprising *α* catalytic subunit and two regulatory subunits (*β* and *γ*), is a crucial cellular energy sensor in most eukaryotic cells [12]. Energy depletion conditions activate AMPK which promotes ATP production by stimulating catabolic processes and increasing some glycolytic genes such as PFKFB3 and PFKFB4 [13, 14] and inhibits anabolic pathways requiring ATP such as gluconeogenesis, protein synthesis, cholesterol synthesis, and triglycerides synthesis [15–18]. In addition, AMPK receives hormonal and nutritional signals in the hypothalamus and maintains energy homeostasis [19] by regulating feeding behavior [20], circadian rhythms [21], and energy expenditure [22, 23]. This review provides a general concept on conditions that activate and inhibit hypothalamic AMPK activity and the physiological effects resulting from increased AMPK activity.

## 2. AMP-Activated Protein Kinase: Structure and Regulation

### 2.1. Structure

AMPK is a heterotrimeric serine/threonine kinase expressed in different tissues including the brain, liver, and skeletal muscle. AMPK consists of a catalytic *α* subunit, a regulatory *β* subunit, and an AMP/ATP binding *γ* subunit [24–26]. *α* subunit contains an N-terminal kinase domain and a C-terminal regulatory domain required for interaction with *β* and *γ* subunits. *β* subunit associates with *α* and *γ* subunits through the C terminus and binds to oligosaccharides through the glycogen-binding domain also known as the carbohydrate-binding module (CBM) [27]. *γ* subunit isoforms have four tandem repeats called cystathionine-*β*-synthase (CBS) that bind to AMP or ATP molecules forming an adenine nucleotide-binding region [28]. There are 2 or 3 genes encoding each subunit; as a result, there are 12 possible heterotrimeric combinations, with splice variants further increasing the potential diversity [29].

### 2.2. Regulation

**Figure 1:**
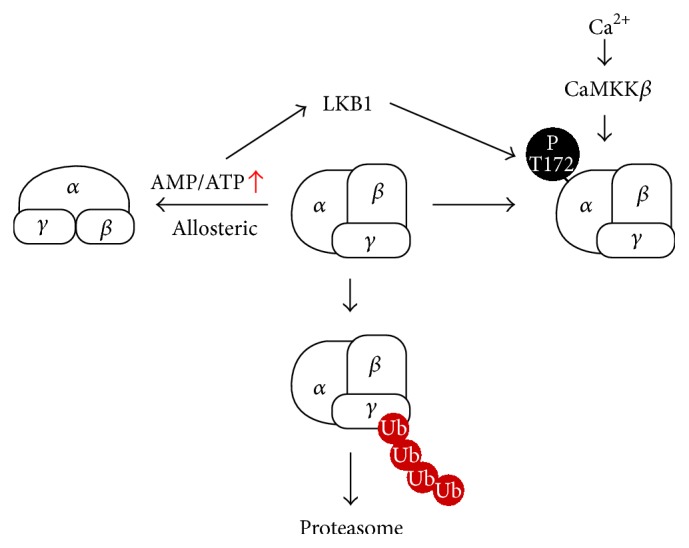
The structure and regulation of AMPK. AMP-activated protein kinase (AMPK) complexes are heterotrimeric kinase composed of *α*, *β*, and *γ* subunits in a 1 : 1 : 1 ratio. AMPK is specifically activated by AMP and its analogues via allosteric activation. The activity of AMPK is also induced through reversible phosphorylation, especially on Thr-172 residue. Calcium and calmodulin increase the phosphorylation of AMPK through Ca^2+^/calmodulin-dependent protein kinase kinase *β* (CaMKK*β*) while the elevated AMP : ATP ratio can enhance liver kinase B1 (LKB1) activity. AMPK activation is also regulated by the ubiquitin proteasome system.

AMPK activity is determined by the AMP : ATP ratio. It can also be regulated by AMP's direct allosteric activation, reversible phosphorylation, ADP, or degradation by ubiquitination (Figure 1). The allosteric activation of AMPK is specifically affected by AMP and its analogues [30]. AMP can cause more than 10-fold allosteric activation when its concentration is 1-2 times lower than ATP. Besides that, AMP also enhances the activation of AMPK by inhibiting Thr-172 dephosphorylation (10-fold more potent than ADP) and only AMP enhances liver kinase B1- (LKB1-) induced Thr-172 phosphorylation [31]. AMP allosteric control was previously thought to be mediated by both *α* and *γ* subunits but one study pointed out that the degree of activation is affected by the nature of *γ* isoform in the AMPK complex [26]. However, the highest allosteric activation achieved is approximately 5-fold whereas the effect of phosphorylation on AMPK activity can be much higher [32].

Phosphorylation is the primary regulator of AMPK activity. AMPK requires the phosphorylation on a threonine residue (Thr-172) within the catalytic subunit for its activation [33]. Among the kinases involved in the phosphorylation of AMPK, a lot of research has focused on the two upstream kinases, Ca^2+^/calmodulin-dependent protein kinase kinase *β* (CaMKK*β*) and the tumor suppressor kinase, liver kinase B1 (LKB1) [34–37]. CaMKK*β*, activated by calcium and calmodulin, is known to increase the phosphorylation of AMPK at Thr-172 [38] while LKB1 is the major kinase for AMPK's phosphorylation under energy stress conditions [39]. Besides Thr-172, many phosphorylation sites have been identified within *α* and *β* subunits of AMPK but, for most of these sites, the direct effects on AMPK activation and physiological relevance are still unclear [32].

Many compounds are known as AMPK activators through an indirect manner: via increasing the intracellular AMP : ATP ratio or via activation or upregulation of upstream kinases. Among these compounds, there are a number of natural compounds involved in the activation of AMPK such as biguanides [40], gallic acid [41], resveratrol [42], berberine [43], baicalein [44], quercetin [45], arctigenin [46], genistein and capsaicin [47], and curcumin [48]. Besides natural compounds, thiazolidinediones, via altering the level of adenine nucleotides, also activate AMPK [49]. Additionally, 5-aminoimidazole-4-carboxamide ribonucleotide (AICAR), a representative AMP analog, is metabolized in cells to 5-aminoimidazole-4-carboxamide-1*β*-D-ribofuranosyl-5′-monophosphate (ZMP), an AMP mimetic, therefore increasing AMP : ATP ratio and activating AMPK [50].

Although ADP does not allosterically activate the enzyme, ADP can protect AMPK from dephosphorylation [51]. Moreover, ADP and AMP can bind to the three *γ* subunit complexes preventing AMPK dephosphorylation at Thr-172. ADP and AMP have different binding abilities on the three *γ* subunits. On *γ*2 complexes ADP and AMP have the same binding potential, while ADP is less potent than AMP on *γ*1 and *γ*3 complexes [52]. ADP can also promote phosphorylation of Thr-172 and, similar to AMP, it requires N-terminal myristoylation of *β* subunit [53].

Recent studies identified the ubiquitin proteasome system as a factor regulating AMPK activity directly and indirectly [54, 55]. AMPK-related kinases, such as AMPK-related kinase 5 (NUAK1) and microtubule-affinity-regulating kinase 4 (MARK4), are polyubiquitinated* in vivo* and interact with the deubiquitinating enzyme ubiquitin specific protease-9 (USP9X). The study provides the first evidence that AMPK family kinases are regulated by polyubiquitin chains [56]. Another study described the suppression of AMPK through ubiquitination and degradation by the cancer-specific MAGE-A3/6-TRIM28 ubiquitin ligase [57]. Additionally, CIDEA which is highly expressed in brown adipose tissue (BAT) has been shown to interact with the regulatory *β* subunit of AMPK resulting in the ubiquitination and degradation of AMPK. Further CIDEA knockout mice showed an increase in stability and activity of AMPK in BAT [58]. Furthermore, malfunction of the ubiquitin/protease system could contribute to energy homeostasis imbalance by increasing inflammation and apoptosis of hypothalamic neurons important for energy homeostasis regulation [59]. In line with this, diet-induced obesity suppresses hypothalamic AMPK activity [60]. These studies indicate the role of ubiquitin/proteasome system in the regulation of AMPK in the hypothalamus.

## 3. The Activators and Inhibitors of AMPK in the Brain

**Figure 2:**
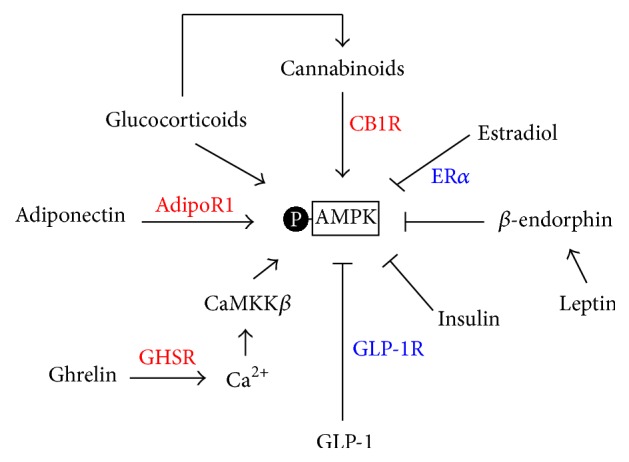
Factors modulating hypothalamic AMPK activity. By inducing AMPK phosphorylation, adiponectin, ghrelin, cannabinoids, and glucocorticoids activate hypothalamic AMPK activity. On the other hand, estradiol, leptin, insulin, and glucagon-like peptide-1 (GLP-1) inhibit central AMPK activity by decreasing AMPK phosphorylation. AdipoR1, adiponectin receptor 1; GHSR, growth hormone secretagogue receptor; CaMKK*β*, Ca^2+^/calmodulin-dependent protein kinase kinase *β*; CB1R, cannabinoid type 1 receptor; ER*α*, estrogen receptor *α*; GLP-1R, glucagon-like peptide-1 receptor.

There are many factors affecting energy intake and energy expenditure via the activation or inhibition of AMPK in the brain, especially in the hypothalamus. They are also variable molecular mechanisms regulating AMPK activity but, in general, these factors affect the activity of AMPK by altering its phosphorylation. In the following, we discuss some factors involved in the activation and inhibition of hypothalamic AMPK activity (Figure 2).

## 4. The Activators

### 4.1. Adiponectin

Adiponectin is secreted from white adipose tissue and plays a role in the central and peripheral regulation of energy homeostasis [61–63]. Adiponectin serves as a starvation signal and regulates feeding behavior by stimulating the phosphorylation of AMPK. In fasting condition, adiponectin increases and stimulates the activity of AMPK in the ARC, leading to induction of food intake and reduction of energy expenditure. After refeeding, decrease in adiponectin levels is accompanied by blunted AMPK activity [64–66]. Of the two adiponectin receptors AdipoR1 and AdipoR2 expressed in the ARC, adiponectin increases AMPK activity and stimulates food intake via AdipoR1. The level of adiponectin and expression of AdipoR1 are elevated in the serum and cerebrospinal fluid during fasting and decrease after refeeding [67]. Acute adiponectin ICV injection was shown to increase the phosphorylation of AMPK [68] while adiponectin deficiency and inhibition of AdipoR1 by adeno-AdipoR1 siRNA suppressed phosphorylation of AMPK in the ARC [67, 69]. The adiponectin/AdipoR1/AMPK pathway has also been implicated in the thiazolidinediones (TZDs) induced body weight gain. TZDs are prescribed for glycemic control but induce body weight gain as a side effect [70, 71]. Pioglitazone treatment was reported to increase food intake and decrease energy expenditure by enhancing the adiponectin signaling and increasing the phosphorylation of AMPK via the AdipoR1 in the hypothalamus [72].

### 4.2. Ghrelin

Ghrelin is a peptide produced mainly by the oxyntic cells of the stomach and released during fasting condition. It is a natural ligand for the hypothalamic growth hormone secretagogue receptor (GHSR) [73]. Ghrelin is the first circulating hormone demonstrated to induce food intake in man [74]. Intracerebroventricular (ICV) and intraperitoneal (IP) administration of ghrelin was shown to stimulate food intake in rats [75]. In the CNS, ghrelin mainly acts on the hypothalamus to regulate appetite and hence energy consumption [76]. ICV administration of ghrelin in rats activates AMPK in both the ventromedial nucleus of the hypothalamus (VMH) [77] and ARC [78] via GHS-R1a [79]. These receptors are mainly expressed in the ARC and the VMH in the hypothalamus [80] and are necessary for ghrelin's effect on hypothalamic AMPK activity. Ghrelin binds to GHSR and activates heterotrimeric G protein containing Gq (also known as G11) leading to increased intracellular Ca^2+^ release [81]. Ghrelin also stimulates the release of Ca^2+^ from ryanodine-sensitive internal stores [82, 83]. Increased intracellular Ca^2+^ activates CaMKK*β* pathway and induces AMPK phosphorylation.

### 4.3. Cannabinoids

Cannabinoids are a class of diverse chemical compounds that act on cannabinoid receptors repressing neurotransmitter release in the brain. Ligands for these receptor proteins include endocannabinoids, phytocannabinoids, and synthetic cannabinoids. Endocannabinoids can stimulate appetite in the hypothalamus via the presynaptic cannabinoid type 1 (CB1) receptor [84]. Cannabinoid receptor antagonists have been reported to suppress the motivation to eat and reduce the consumption of palatable foods. For example, chronic treatment of rimonabant, a CB1 receptor antagonist, resulted in a marked and sustained decrease in body weight and improved metabolic profile [85, 86]. Cannabinoids, just like ghrelin, can stimulate AMPK activity in the hypothalamus leading to increased appetite [87]. Further, androgens such as testosterone induce hyperphagia and potentiate cannabinoid tone at the CB1 receptors by activating AMPK [88]. Endocannabinoids have been shown to be essential for the orexigenic and anorectic effects of ghrelin and leptin, respectively. Acute leptin treatment in normal rats and* ob/ob* mice reduces endocannabinoids level in the hypothalamus indicating the involvement of endocannabinoids in the leptin induced food intake regulation in the hypothalamus [89]. Ghrelin treatment failed to induce orexigenic effect in CB1 receptor knockout mice. In addition, genetic and pharmacological blockade of CB1 receptor inhibited the effects of ghrelin on AMPK activity indicating that the stimulatory effects of ghrelin need an intact cannabinoids signaling pathway for AMPK activity and food intake [79, 90].

### 4.4. Glucocorticoids

Glucocorticoids are potent anti-inflammatory agents used in a wide range of inflammatory and immunologically mediated disease processes. Therapeutic doses of glucocorticoids induce obesity by acting directly or indirectly in the central nervous system to regulate appetite and increase energy intake [91]. Glucocorticoids treatment stimulate AMPK activity in rats' hypothalamus directly [92] or via the induction of endocannabinoid synthesis [93]. Additionally, CB1 knockout mice treated with corticosterone showed an increase in hypothalamic AMPK activity accompanied by blunted weight gain indicating the importance of the CB1 receptor in the glucocorticoid induced activation of the hypothalamic AMPK activity [94]. Further, glucocorticoids upregulate gene expression of the orexigenic NPY as well as AgRP via AMPK phosphorylation at Thr-172 in the arcuate nucleus suggesting that glucocorticoids are essential for the hypothalamic AMPK mediated energy homeostasis [95, 96].

### 4.5. Hypoglycemia

Glucose is the main source of energy source for the body and is particularly essential for normal brain activity. Hypoglycemia, a condition in which the blood glucose drops below normal levels, poses a great danger to the stability and functioning of the brain. The body has therefore developed many mechanisms to prevent glucose insufficiency especially in the brain. Activation of AMPK which senses the nutritional status of the body is among these mechanisms [97]. To demonstrate the role of AMPK as a glucose sensor, ICV administration of glucose reduced hypothalamic AMPK activity while 2-deoxyglucose, an inhibitor of intracellular glucose utilization, induced hypothalamic AMPK activity after 60 minutes of injection [98, 99]. Moreover, selective downregulation of AMPK in the VMH caused impaired response to acute hypoglycemia by glucagon and epinephrine [100] whereas local VMH application of AICAR during hypoglycemia amplified both glucagon and epinephrine levels [101].

## 5. The Inhibitors

### 5.1. Estradiol

Estradiol, or 17*β*-estradiol, is a steroid and estrogen sex hormone. It plays a fundamental role in the reproductive, cardiovascular, skeletal, and central nervous systems. Many studies have shown a correlation between estradiol and metabolic syndromes [102]. In the brain, estradiol receptor (ER) *α* and/or ER*β* are expressed in several hypothalamic nuclei responding to feeding behavior. ER*α* is mainly expressed in POMC neurons while both ER*α* and ER*β* are present in NPY neurons [103, 104]. A study demonstrates that the central action of estradiol in the ventromedial nucleus of the hypothalamus inhibits the activity of AMPK, leading to decreased food intake and increased energy expenditure through the sympathetic nervous system in a feeding-independent manner [105]. Moreover, ovariectomized rats showed increased phosphorylation of hypothalamic AMPK*α* and this effect was reversed after administration of estradiol. In addition, treatment with compound C, an AMPK*α* inhibitor, for 1 week reduced food intake, body weight, plasma leptin, and adiponectin levels [106]. Furthermore, estradiol also regulates AMPK activity in caudal hindbrain A2 noradrenergic neurons [107] and hypothalamic astrocyte pAMPK is augmented by hypoglycemia in the presence of estradiol [108]. In addition, there is a reduction of hypothalamic AMPK depending on estradiol levels in pregnant rats [109].

### 5.2. Leptin

Leptin is a hormone secreted by adipocytes and is essential for food intake and energy expenditure regulation. In the skeletal muscle, leptin increases AMPK activity directly as well as indirectly through stimulation of the hypothalamosympathetic axis. On the other hand, in the hypothalamus, leptin decreases AMPK activity [110].* In vivo* administration of leptin decreases hypothalamic AMPK activity leading to suppressed feeding behavior [111]. In addition, leptin also inhibits AMPK activity in the ARC and PVH exerting its effects on food intake and body weight [20]. To investigate the role of AMPK activity in sympathetic effects of leptin* in vivo*, siRNA was administered to knock down AMPK*α*2 in rats. As a result, leptin effects on body weight, food intake, and blood FFA levels were diminished in AMPK*α*2 siRNA-treated rats [112]. Leptin regulates food intake by selectively activating POMC neurons. Leptin binds to leptin receptor and directly depolarizes the POMC neurons stimulating *β*-endorphin and *α*-melanocyte-stimulating hormone (*α*-MSH) secretion to downregulate elevated synaptic activity [113]. After that, leptin and opioids from POMC can inhibit the activation of AMPK on a positive feedback loop [83].

### 5.3. Insulin

Insulin is a hormone produced by beta cells in the pancreas. It regulates the metabolism of carbohydrates and fats by promoting the absorption of glucose from the blood. In the central nervous system, insulin acts as a potent anorexigenic hormone [114]. Insulin can reduce the activity of *α*2-AMPK by 25–40% in all hypothalamic regions but not in the cortex and the effects of insulin are more widespread in the hypothalamus than that of leptin [20]. Furthermore, ICV injection of an acute dose of taurine activating insulin pathway through AKT/FoxO1 reduces food intake and locomotor activity by suppressing AMPK activity via the mTORC1 pathway [115]. Insulin resistance is described as the inability of insulin to regulate blood glucose level and usually leads to hyperglycemia accompanied by metabolic syndrome development including obesity and diabetes. In diabetic rat, hypothalamic AMPK phosphorylation and *α*2-AMPK activity are higher. Moreover, chronic insulin treatment or suppression of hypothalamic AMPK activity completely prevents diabetes-induced changes in food intake as well as in hypothalamic AMPK activity [116]. In addition, the effect of insulin is significant in the ARC/VMH and paraventricular nucleus (PVN) [117].

### 5.4. Glucagon-Like Peptide-1 (GLP-1)

GLP-1 is a neuropeptide and an incretin hormone released by L cells in the ileum and colon. In the central nervous system, there are many neuronal populations expressing GLP-1 and GLP-1 receptors (GLP-1R) especially in the hypothalamic nuclei which is important for energy homeostasis [118]. In the hypothalamus, ICV GLP-1 administration inhibits feeding in fasted rats, demonstrating that GLP-1 is a physiological mediator of satiety [119]. In addition, a reduction in the CNS GLP-1 neuronal activity during food deprivation may act to induce feeding behavior and ICV leptin administration prevents a decrease in hypothalamic GLP-1 peptide content [120]. GLP-1 decreases feeding behavior through the inhibition of AMPK activity. GLP-1 treatment can inhibit the activities of AMPK and p70S6K, the downstream target of mTOR signaling, even at the maximal activity of these protein kinases in the ventromedial and lateral hypothalamic areas [121]. GLP-1 and leptin can act together to partly reduce feeding behavior by inhibiting AMPK after binding of the POMC derivative *α*-MSH to its receptor melanocortin-4-receptor (MC4-R) [122]. Moreover, the GLP-1R agonist, liraglutide, was shown to dephosphorylate and hence inactivate AMPK leading to increased energy expenditure [123]. The GLP-1 receptors in the ventromedial nucleus of the hypothalamus are also essential for thermogenesis in brown adipose tissue and for the browning effect of white adipose tissue to increase energy expenditure and both effects are partly mediated through the inhibition of AMPK activity [124].

## 6. Physical Exercise and AMPK Activation

AMPK is stimulated by contractile activity in the skeletal muscles and is therefore regarded as an important factor in the regulation of cell metabolism in exercise-induced changes in muscle glucose and fatty acid metabolism. Indeed, high intensity exercise was shown to significantly increase the activity of *α*2-AMPK but not *α*1-AMPK in healthy human subjects suggesting that *α*2-AMPK might play an important role in metabolic responses to exercise in skeletal muscles [125]. Whereas the role of exercise in the central nervous system remains to be clearly elucidated, the effect of exercise in inducing appetite suggests that exercise and physical activities might somehow influence the activity of AMPK in the hypothalamus. However, there was no significant change observed in the hypothalamic AMPK activity after 1 h of strenuous exercise in rats despite an increase in plasma ghrelin [126]. On the contrary, AMPK activity in the hypothalamus was reduced after leptin infusion in both lean and diet-induced obese rats after acute exercise. In addition, exercise was shown to improve insulin and leptin signaling in the cerebral cortex and hypothalamus in diabetic rats treated with high dose of dexamethasone [127]. This indicates that the effects of leptin on AMPK activity, potentiated by acute exercise, may contribute to appetite suppression in the hypothalamus [128].

## 7. The Physiological Effects of Hypothalamic AMPK

**Figure 3:**
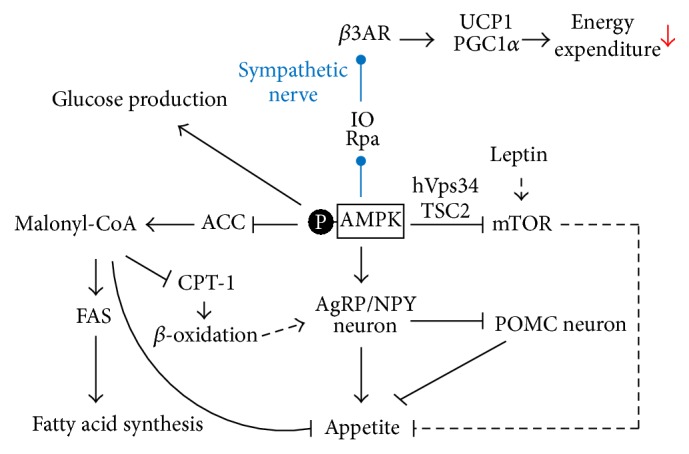
The physiological effects of hypothalamic AMPK. Activated in energy depletion conditions, hypothalamic AMP-activated protein kinase (AMPK) restores energy homeostasis by promoting appetite and reducing energy output. Activated hypothalamic AMPK stimulates the orexigenic neuropeptides leading to enhanced food intake and inhibits anorexigenic neuropeptide suppressing food intake. The mammalian target of rapamycin (mTOR), suppressed by the activation of AMPK, also decreases feeding behavior under the effect of leptin. AMPK activity can induce appetite via the inhibition of malonyl-CoA and activation of carnitine palmitoyltransferase- (CPT-) 1. The inhibition of AMPK on malonyl-CoA can lead to decreased fatty acid synthesis and increased *β*-oxidation. Furthermore, increased *β*-oxidation could result in the induction of orexigenic gene expression. Besides that, AMPK activation through the sympathetic nerve can reduce thermogenesis and decrease energy expenditure. Additionally, activated hypothalamic AMPK can lead to enhanced glucose production. AgRP, agouti-related protein; NPY, neuropeptide Y; POMC, proopiomelanocortin; TSC2, tuberous sclerosis complex 2; hVps34, mammalian vacuolar protein sorting 34 homologue; ACC, acetyl-CoA carboxylase; FAS, fatty acid synthase; Rpa, raphe pallidus; IO, inferior olive; *β*3AR, *β*3-adrenergic receptor; UCP1, uncoupling protein 1; PGC1*α*, peroxisome proliferator-activated receptor-gamma coactivator 1 alpha.

AMPK plays an important role as a nutrient and energy sensor in the central nervous system and maintains energy homeostasis by regulating feeding behavior and energy expenditure (Figure 3).

### 7.1. Feeding Behavior

Hypothalamic AMPK is considered a key player in the regulation of feeding behavior and energy balance due to its role in sensing and responding to hormonal and nutritional signals from the peripheral. Moreover, studies on knockout mice lacking *α*2-AMPK in POMC and AgRP neurons indicated that AMPK is essential for energy homeostasis regulation and glucose sensing by POMC and AgRP neurons. Specifically, the *α*2-AMPK POMC knockout mice developed obesity due to reduced energy expenditure and dysregulated food intake while *α*2-AMPK AgRP mice showed a lean phenotype. Interestingly, both knockout groups remained sensitive to leptin [129]. Regarded as a possible explanation, a report has suggested that nutrients signals do not regulate the NPY/AgRP or POMC neurons themselves but affect a presynaptic positive feedback loop involving AMPK and create excitatory or inhibitory synaptic input [83]. Although the downstream target(s) of AMPK remain unclear in the presynaptic neurons, AMPK acts as a mediator when activated in fasting state following Ca^2+^ influx and consequent neurotransmitter release into the NPY/AgRP neurons leading to the activation of these neurons. As a result, there is an increase in appetite and feeding behavior. Furthermore, the activation of orexigenic signals also inhibits anorexigenic signals [130, 131].

In discrete hypothalamic regions, fatty acid metabolism acts as a sensor for nutrient availability [132–134]. Among the many factors involved in fatty acid metabolism, malonyl-CoA is regarded as a potential candidate for food intake regulation. Malonyl-CoA is involved in both fatty acid synthesis and fatty acid *β*-oxidation. By inhibiting mitochondrial carnitine palmitoyltransferase- (CPT-) 1, malonyl-CoA reduces the number of fatty acid-CoA shuttled into the mitochondria for *β*-oxidation. Besides that, it is also an important substrate for fatty acid synthesis. The level of hypothalamic malonyl-CoA is low in fasted condition and rapidly increases on refeeding [135]. Moreover, induction of malonyl-CoA and inhibition of hypothalamic CPT-1 leads to decreased food intake whereas reduction in malonyl-CoA increases feeding behavior and weight gain [136–138]. Interestingly, AMPK activation leads to phosphorylation and inactivation of the enzyme acetyl-CoA carboxylase (ACC), which catalyzes the carboxylation of acetyl-CoA to produce malonyl-CoA, resulting in decreased hypothalamic malonyl-CoA and/or long-chain fatty acid-CoA and inducing fatty acid oxidation and feeding behavior. Furthermore, brain-specific CPT-1c knockout mice exhibit decreased food intake and lower body weight compared to wild-type littermates [139] while increased CPT-1c by activation of AMPK leads to the induction of hypothalamic ceramide synthesis in the endoplasmic reticulum [140]. Besides that, activated AMPK also induces CPT-1a in the mitochondria [77] and the induction of both CTP-1 isoforms results in increasing mRNA expression of* AgRP* and* NPY* genes by the activation of brain-specific homeobox transcription factor (BSX) together with the forkhead box protein O1 (FoxO1) and the phosphorylated cyclic adenosine monophosphate response-element binding protein (pCREB) [141]. Additionally, AMPK suppresses the synthesis of ACC, fatty acid synthase (FAS), and other enzymes for lipid biogenesis in many tissues, including the hypothalamus, by inhibiting expression of the transcription factor sterol-regulatory-element-binding transcription factor 1 (SREBP1c) [142, 143].

Besides AMPK, the mammalian target of rapamycin (mTOR) is also regarded as a hypothalamic regulator for food intake. mTOR signaling responds to nutrients availability and colocalizes with NPY/AgRP and POMC/cocaine- and amphetamine-regulated transcript (CART) neurons in the arcuate nucleus. Leptin induces hypothalamic mTOR activity in satiety condition and the inhibition of mTOR signaling blunts leptin's anorectic effect [144]. Furthermore, mTOR is inhibited by AMPK-dependent mechanisms* in vitro* via tuberous sclerosis complex 2 (TSC2) and mammalian vacuolar protein sorting 34 homologue (hVps34) [145, 146]. On the contrary, AMPK is also a substrate for mTOR-p70S6 kinase and is phosphorylated at Ser-491 [147]. Moreover, in a high-protein diet-induced weight loss, both AMPK and mTOR are modulated in the same specific neuronal subsets and regulates hypothalamic neuropeptides, indicating overlapping localization and function [148]. Thus, the overlap in hypothalamic mTOR and AMPK and can lead to a reciprocal interaction in regulating feeding behavior.

### 7.2. Energy Expenditure

Food is the main source of energy utilized by the body for various physiological functions and excess energy is stored as fat or released from the body in form of heat. Total energy expenditure can be subdivided into three principal components: obligatory energy expenditure; energy expenditure resulting from physical activity and expenditure attributed to adaptive thermogenesis defined as heat production in response to environmental temperature or diet [149]. Changes in the environment or diet condition can be detected and regulated by the brain, especially the hypothalamus. Among the many brain regions involved in thermoregulation, the ventromedial nucleus of the hypothalamus, VMH, was the first to be identified [150]. Electrophysiological stimulation of the VMH enhances interscapular BAT temperature in both lean and obese rats [151]. Additionally, when stimulated, VMH elicits an increase in the rate of norepinephrine turnover in BAT and this effect is abrogated by sympathetic ganglion blockade [152–154]. Furthermore, VMH-specific steroidogenic factor 1 (SF-1) knockout mice display impaired thermogenesis and decreased gene expression of uncoupling protein 1 (UCP1) and peroxisome proliferator-activated receptor-gamma coactivator 1 alpha (PGC1*α*) [155]. On the other hand, mice lacking forkhead box protein O1 (FoxO1) in SF-1 neurons of the VMH have a lean phenotype due to increased thermogenesis [156]. These studies indicate the role of VMH in thermoregulation. Additionally, sympathetic outflow to tissues involved in thermoregulation and metabolism is regulated by central pathways, including neurons in raphe pallidus (Rpa) and inferior olive (IO) [157–159].

Recent studies have indicated the role of hypothalamic AMPK in sympathetically activated BAT thermogenesis. Constitutively active AMPK*α* (AMPK*α*-CA) overexpression is associated with a specific reduction in the expression of BAT thermogenic markers whereas dominant-negative AMPK*α* (AMPK*α*-DN) promotes expression of these markers. This indicates that AMPK in the VMH modulates BAT thermogenesis via the sympathetic nervous system [160]. Furthermore, bone morphogenetic proteins (BMPs) knockout mice exhibit thermogenic impairment and display changes in neuropeptide levels and reduced phosphorylation of AMPK. Central BMP8B treatment increases hypothalamic AMPK-dependent sympathetic activation of BAT [161]. Besides BMPs, estradiol and GLP-1 also regulate BAT thermogenesis via hypothalamic AMPK. Estradiol inhibits AMPK activity in the VMH via estrogen receptor alpha, resulting in increased expression of BAT thermogenic markers through a feeding-independent manner [105]. GLP-1R agonist, liraglutide, also stimulates BAT thermogenesis and this central liraglutide-induced thermogenesis is blunted by activation of hypothalamic AMPK [123]. In addition, some compounds like thiazolidinediones and nicotine also modulate hypothalamic AMPK to induce BAT thermogenesis through the sympathetic nervous system [72, 162].

### 7.3. Glucose Homeostasis

AMPK plays a fundamental role in energy homeostasis as a glucose sensor to protect the CNS from hypoglycemic condition. One of the most important reactions of AMPK towards hypoglycemia is the counterregulatory hormonal responses [99] through glucagon, epinephrine, and corticosterone [100, 101, 117]. Hypothalamic AMPK activation is sufficient and necessary for altering glucose production* in vivo*. Inhibition of hypothalamic AMPK significantly decreases glucose production with no changes in peripheral glucose uptake during the hyperinsulinemic-euglycemic clamps [163]. Additionally, deletion of LKB1, an important kinase upstream of AMPK, in POMC neurons leads to an impairment in peripheral glucose homeostasis [36]. Furthermore, central administration of olanzapine activates hypothalamic AMPK resulting in induction hepatic glucose production via the sympathetic nervous system [164].

## 8. Concluding Remarks

AMPK has recently been identified as a key regulator for hypothalamic functions in balancing energy homeostasis. Targeted by nutritional signals and hormones, AMPK activation in hypothalamus, via phosphorylation mainly at Thr-172, leads to changes in both feeding behavior and energy expenditure to protect the body against energy depletion. Recent studies have shown that AMPK, in both central and peripheral region, plays an important role in the activation mechanism of metformin, thiazolidinediones, and statins [72, 165–167]. Moreover, considering the role of hypothalamic AMPK in lipid and glucose metabolism, central AMPK specific drugs can be developed for treatment of metabolic syndrome. Thus, hypothalamic AMPK should be considered as a potential therapeutic target for metabolic diseases.

Although hypothalamic AMPK acts as a regulator for energy status, the role of central AMPK in responding to exercise activities remains unclear and this warrants more studies to investigate its role, if any, and the underlying molecular mechanisms.
